# Localization of reelin signaling pathway components in murine midbrain and striatum

**DOI:** 10.1007/s00441-014-2022-6

**Published:** 2014-11-25

**Authors:** Ahmed Sharaf, Belal Rahhal, Björn Spittau, Eleni Roussa

**Affiliations:** 1 Institute for Anatomy and Cell Biology, Department of Molecular Embryology, Albert-Ludwigs-University Freiburg, Albertstrasse 17, 79104 Freiburg, Germany; 2 Physiology Department, Faculty of Medicine and Health Sciences, An-Najah Natinal University, Nablus, Palestine; 3 Institute for Anatomy and Cell Biology, Department of Neuroanatomy, Albert-Ludwigs-University Freiburg, Freiburg, Germany; 4 Department of Histology and Cytology, Faculty of Veterinary Medicine, Zagazig University, Zagazig, 44519 Egypt

**Keywords:** Reelin, ApoER2, Vldlr, Dab1, Mesencephalic dopaminergic neurons, Striatal neurons, Mouse

## Abstract

We investigated the distribution patterns of the extracellular matrix protein Reelin and of crucial Reelin signaling components in murine midbrain and striatum. The cellular distribution of the Reelin receptors VLDLr and ApoER2, the intracellular downstream mediator Dab1, and the alternative Reelin receptor APP were analyzed at embryonic day 16, at postnatal stage 15 (P15), and in 3-month-old mice. Reelin was expressed intracellularly and extracellularly in midbrain mesencephalic dopaminergic (mDA) neurons of newborns. In the striatum, Calbindin D-28k^+^ neurons exhibited Reelin intracellularly at E16 and extracellularly at P15 and 3 months. ApoER2 and VLDLr were expressed in mDA neurons at E16 and P15 and in oligodendrocytes at 3 months, whereas Dab1 and APP immunoreactivity was observed in mDA at all stages analyzed. In the striatum, Calbindin D-28k^+^/GAD67^+^ inhibitory neurons expressed VLDLr, ApoER2, and Dab1 at P15, but only Dab1 at E16 and 3 months. APP was always expressed in mouse striatum in which it colocalized with Calbindin D-28k. Our data underline the importance of Reelin signalling during embryonic development and early postnatal maturation of the mesostriatal and mesocorticolimbic system, and suggest that the striatum and not the midbrain is the primary source of Reelin for midbrain neurons. The loss of ApoER2 and VLDLr expression in the mature midbrain and striatum implies that Reelin functions are restricted to migratory events and early postnatal maturation and are dispensable for the maintenance of dopaminergic neurons.

## Introduction

Reelin is an extracellular matrix protein with crucial roles during neurodevelopment and adult synaptic plasticity. In the developing brain, Reelin is produced and secreted by Cajal-Retzius cells that reside in the marginal zone (Del Río et al. 1997) and regulates normal migration and the formation of laminar brain structures including the cortex, hippocampus, and cerebellum (D’Arcangelo et al. 1999; Hiesberger et al. 1999; Curran and D’Arcangelo 1998; Tissir and Goffinet 2003; Förster et al. 2006). Reelin is localized in specific neuronal populations (Lacor et al. 2000) in both adult and embryonic rodent brains and exerts its effects through binding to apolipoprotein E receptor type 2 (ApoER2) and very low density lipoprotein receptors (VLDLr; Jossin et al. 2004; D’Arcangelo et al. 1997, 1999; Morimura et al. 2005). Binding of Reelin to these receptors causes clustering of the receptors and the activation of tyrosine kinase, which in turn phosphorylates the cytoplasmic disabled adaptor protein (Dab1; D’Arcangelo et al. 1999; Hiesberger et al. 1999). Upon phosphorylation of Dab1, Src-family tyrosine kinases and other non-receptor tyrosine kinases become activated and trigger multiple downstream signaling cascades (Arnaud et al. 2003; Jossin et al. 2003; Howell et al. 1999), ultimately leading to the regulation of neuronal migration and neurite outgrowth (Del Río et al. 1997; Borrell et al. 1999; Niu et al. 2004; Beffert et al. 2005; Hiesberger et al. 1999; Arnaud et al. 2003).

Disruptions of the Reelin signaling pathway lead to severe motor deficits. Mice deficient for Dab1 and for both ApoER2/VLDLr display the same migratory phenotypes and are similar to *reeler* mice (Sharaf et al. 2013; Herz and Bock 2002; Trommsdorff et al. 1999). The migratory deficits in *reeler* mice might be attributable to the direct effect of Reelin on the neurons and/or on the differentiation of radial glia cells, which have an important role in controlling neuronal migration (Förster et al. 2002). In addition, Reelin- and Dab1-deficient mice show deficits in the normal migration of mesencephalic dopaminergic neurons (mDA; Kang et al. 2010) and hindbrain motor neurons (Rossel et al. 2005).

In the mature brain, numerous studies have demonstrated the role of Reelin in synaptic plasticity. Accordingly, ApoER2, VLDLr, and Dab1 remain expressed in the adult brain. Interestingly, Reelin can also bind to other transmembrane protein receptors, including amyloid beta precursor proteins (APP) in vivo and in vitro (Hoe et al. 2009). The biological significance of the Reelin/APP interaction is not yet elucidated but, during the last few years, accumulating evidence has suggested the involvement of Reelin in the pathogenesis of Alzheimer’s disease. Reelin is indeed downregulated in APP-overexpressing mice but is upregulated in APP-deficient mice (Hoe et al. 2009).

mDA neurons are divided into three subpopulations: the substantia nigra pars compacta (SNpc; A9), the ventral tegmental area (VTA; A10), and the retrorubral field (RrF; A8). With regard to their connectivity and morphology, mDA neurons can be separated into two subpopulations: the calbindin-expressing mDA neurons that innervate ventral striatal, limbic, and cortical areas, and the GIRK2-positive (GIRK2^+^) mDA neurons that project to the striatum (Björklund and Dunnett 2007).

We have previously described the roles of ApoER2 and VLDLr in the proper migration and positioning of mouse mDA neurons (Sharaf et al. 2013). VLDLr- and ApoER2-mutant mice exhibit both a reduction in and abnormal positioning of mDA neurons, and ApoER2/VLDLr double-knockout mice show a phenotype comparable with the phenotypes observed for Reelin- and Dab1-mutant mice, demonstrating the essential roles of ApoER2 and VLDLr in the Reelin-mediated migration and positioning of mDA neurons. However, the presence and distribution of Reelin signaling components in the mature dopaminergic system has not yet been addressed. In the present study, we have investigated the distribution patterns of Reelin signaling pathway components in the murine midbrain and striatum during embryonic, postnatal, and adult stages.

## Materials and methods

### Experimental animals

This study was carried out in strict accordance with national health and ethical regulations, and the care of animals was in accordance with institutional guidelines. The protocol was approved by the Committee on the Ethics of Animal Experiments of Freiburg University. Mice were studied at embryonic (E16), postnatal (P0-P15), and adult stages (3 months old). Pregnant mice were killed by cervical translocation, embryos were collected, and brains were immediately dissected and immersion-fixed in a 4 % paraformaldehyde (PFA) solution. Mice at postnatal ages were anesthetized by using 10 % ketamin (20 mg/kg, Pfizer) and 2 % Rumpon (4 mg/kg, Bayer Healthcare) and transcardially perfused with a freshly prepared 4 % PFA solution in phosphate-buffered saline (PBS, pH 7.2; Merck, Germany). Following perfusion, the brains were dissected and postfixed in 4 % PFA for at least 24 h.

### Immunohistochemistry

Mouse brains were fixed in 4 % PFA overnight, followed by fixation in Bouin’s solution for 4 h, and were subsequently embedded in paraffin. Midbrain and striatum were then cut into coronal serial sections (10 μm). For immunohistochemistry, sections were deparaffinized, rehydrated with descending grades of ethanol, and washed with PBS. Antigen retrieval was carried out in 0.1 M citrate buffer (pH 6) for 10 min by using a microwave oven (600 W). Sections were incubated with 10 % H_2_0_2_ in methanol for 15 min to block endogenous peroxidase activity in the tissue. They were then blocked by using PBS containing 10 % normal goat serum (NGS; Life Technologies, Germany) and 10 % Triton X-100 (Sigma, Germany) to minimize nonspecific labeling, incubated with primary antibodies diluted in blocking solution overnight at 4 °C, incubated for 1 h in PBS containing biotinylated goat anti-mouse secondary antibody (1:300; Molecular Probes), washed in PBS, and incubated with an Avidin-Biotin complex (ABC, Vector Labs) for 1 h. Peroxidase reaction products were visualized by using a peroxidase substrate kit (SK 4700, Vector labs) for 10 min as described by Adams (1981). For double-immunohistochemistry, sections were subsequently incubated with goat anti-rabbit IgG conjugated with alkaline phosphatase (1:300; Molecular Probes) for 1 h at room temperature. The alkaline phosphatase reaction was visualized by incubation with fast red tablets (Roche, Germany) dissolved in 0.1 M TRIS-HCl (pH 8.2) for 10–30 min. Sections stained with peroxidase were dehydrated with ascending grades of ethanol, cleared with xylene, and coverslipped by using an Entellan mounting medium (Merck, Germany). Sections labeled with alkaline phosphatase were mounted with Aquatex (Merck, Germany).

### Double-immunofluorescence

For double-immunofluorescence, sections were treated as above and incubated with the primary antibodies in blocking solution overnight at 4 °C. Subsequently, sections were washed in PBS and incubated with goat anti-mouse Alexa Fluor 568 (1:500) and goat anti-rabbit Alexa Fluor 488 (1:500) secondary antibodies (Molecular Probes) for 1 h at room temperature. Sections were washed with PBS and mounted with Fluoromount-G. Slides were then observed under an epifluorescence microscope (Zeiss Axioplan 2, Jena, Germany). Confocal images were captured by using the Leica SP8 confocal imaging system (Leica, Wetzlar, Germany).

### Overview of primary antibodies

The specificity of Reelin, ApoER2, VLDLr, and Dab1 antibodies had previously been tested on brain tissue from mutant mice, and the optimal antibody dilution was individually determined for each antibody. A list of the primary antibodies used in the present study is shown in Table 1.

**Table 1:** List of primary antibodies used (*VLDLr* very low density lipoprotein receptor, *ApoER2* apolipoprotein E receptor type 2, *APP* amyloid beta precursor protein, *GAD* glutamic acid decarboxylase, *NeuN* neuronal nuclear antigen, *TH* tyrosine hydroxylase, *Dab1* disabled adaptor protein, *GFAP* glial fibrillary acidic protein)

Antibody name	Company	Dilution
Anti-Reelin (mouse)	Millipore (MAB5364)	1:300
Anti-VLDLr (rabbit)	Santa Cruz Biotechnology (sc-20745)	1:100
Anti-ApoER2 (rabbit)	Santa Cruz Biotechnology (sc-20746)	1:100
Anti-APP (mouse)	Millipore (MAB348)	1:100
Anti-GAD67 (mouse)	Millipore(MAB5406)	1:500
Anti-Calbindin D-28k (rabbit)	Millipore (AB1778)	1:500
Anti-Calbindin D-28k (mouse)	Sigma-Aldrich (C9848)	1:500
Anti-NeuN (mouse)	Millipore (MAB377)	1:200
Anti-TH (rabbit)	Millipore (AB152)	1:1000
Anti-TH (mouse)	Millipore (MAB318)	1:1000
Anti-Dab1 (mouse)	Provided by Dr. Hans Bock, Klinik for Gastroenterology, University of Duesseldorf	1:500
Anti-Dab1 (rabbit)	Abcam (ab111684)	1:500
Anti-GFAP (mouse)	Millipore (MAB3402)	1:500
Anti-Olig2 (rabbit)	Millipore (AB9610)	1:300
Tomatolectin-fluorescein-isothiocyanate	Sigma-Aldrich (L0401-1MG)	1:200

## Results

### Expression of reelin in mouse midbrain and striatum

**Fig. 1:**
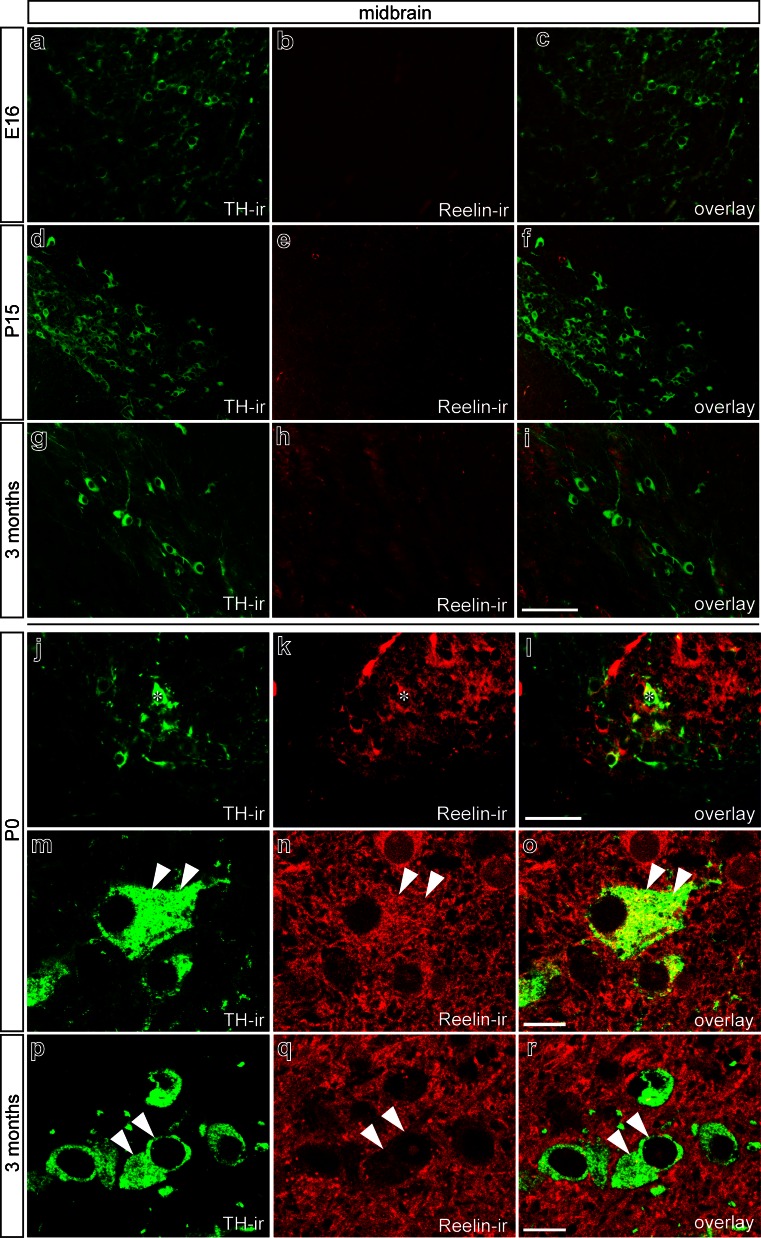
Reelin distribution pattern in mouse midbrain. **a–l** Double-immunofluorescence for tyrosine hydroxylase (*TH*) and Reelin in substantia nigra neurons at embryonic day 16 (*E16*; **a–c**), at postnatal day 15 (*P15*; **d–f**), in newborn (*P0*; **j–l**), and in 3-month-old mice (**g–i**). At E16 (**a–c**), Reelin immunolabeling cannot be detected in dopaminergic neurons. At P15 (**d-f**) and in 3-month-old mice (**g–i**), mesencephalic dopaminergic (mDA) neurons of the substantia nigra are devoid of Reelin immunoreactivity (*Reelin-ir*). However, weak extracellular Reelin immunoreactivity is observed at P15 and 3 months. In newborn mice (P0), TH (**j**) and Reelin (**k**) are co-localized, demonstrating that midbrain neurons (*asterisks*) exhibit an intracellular Reelin distribution. Confocal images demonstrate intracellular (*arrowheads*) Reelin signals in TH^+^ midbrain mDA neurons and an extracellular Reelin localization **m–o**. At 3 months, Reelin immunoreactivity is present extracellularly, whereas TH^+^ neurons (*arrowheads*) show no intracellular Reelin signals (**p–r**). *Bars* 50 μm (**a–l**), 10 μm (**m–r**)

To elucidate the expression patterns of Reelin and its signaling components, including the transmembrane receptors VLDLr, ApoER2, APP, and the cytoplasmic Dab1, in the mouse midbrain and striatum, immunohistochemical studies on coronal brain sections from E16 to 3 months were performed. In order to confirm the specificity of the Reelin antibody, brain sections obtained from Reelin-deficient mice were used as a negative control; no Reelin was detected (data not shown). As demonstrated in Fig. 1a–c, Reelin was not expressed by neurons postive for tyrosine hydroxylase (TH) in the midbrain at E16. Similar results were obtained in midbrain samples from P15 and 3-month-old mice. Whereas no intracellular Reelin signal was detectable at P15 (Fig. 1d–f) and 3 months (Fig.1g–i), extracellular immunoreactivity for Reelin was observed. Interestingly, at P0, distinct Reelin positivity was detected in the cytoplasm of midbrain neurons and in the adjacent extracellular space (Fig. 1j–o) indicating that Reelin expression in the midbrain was restricted to a relatively small time period, and that Reelin was afterwards stored in extracellular pools. Figure 1p–r demonstrates that TH^+^ midbrain neurons at 3 months show virtually no Reelin positivity. However, a distinct Reelin immunoreactivity could be observed in the extracellular space surrounding TH^+^ neurons.

**Fig. 2:**
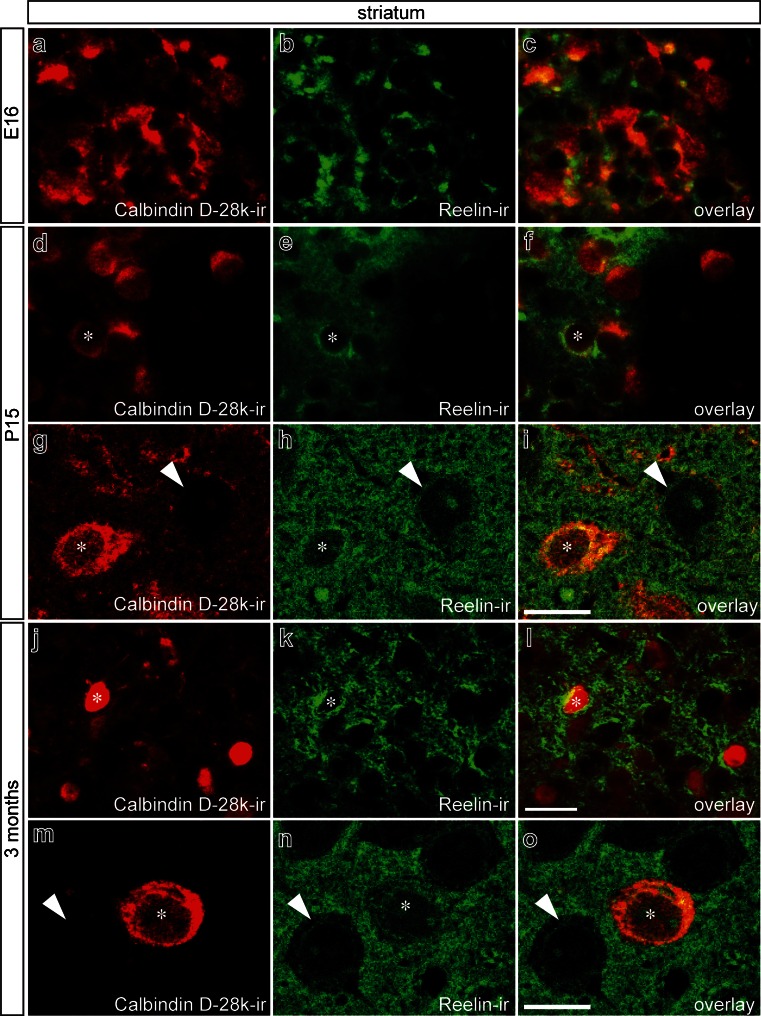
Reelin distribution pattern in mouse striatum. Double-immunofluorescence for Calbindin D-28k and Reelin in mouse striatum at E16 (**a–c**), at P15 (**d–i**), and in 3-month-old mice (**j–o**). At E16, Calbindin D-28k and Reelin co-localize in the cytoplasm in virtually all cells. At P15 (**d–f**), Reelin immunoreactivity is found extracellularly and intracellularly (*asterisks*). Confocal images demonstrate that Calbindin D-28k-positive (Calbindin D-28k^+^) neurons (*asterisks*) and Calbindin D-28k-negative neurons (*arrowheads*) still show cytoplasmic Reelin immunoreactivity (**g–i**). At 3 months, Calbindin D-28k^+^ neurons (*asterisks*) show weak intracellular Reelin immunoreactivity (**j–o**). Moreover, Reelin is deposited extracellularly, and Calbindin D-28k-negative neurons (*arrowheads*) display virtually no Reelin immunoreactivity (**m–o**). *Bars* 20 μm (**a–f**, **j–l**), 10 μm (**g–i**, **m–o**)

Analysis of Reelin expression in the striatum at E16, P15, and 3 months revealed strong Reelin immunoreactivity at all time points examined. At E16, Reelin was found to be located in the cytoplasm of striatal neurons (Fig. 2a–c), most of which were positive for Calbindin D-28k, a marker for inhibitory spiny neurons. At P15, a similar expression pattern of Reelin was observed (Fig. 2d–f); however, only some spiny interneurons (Calbindin D-28k^+^) displayed cytoplasmic Reelin staining (asterisks in Fig. 2d–i). Interestingly, Reelin immunoreactivity was further found in the extracellular space surrounding striatal neurons (Fig. 2g–i). Analysis of striatal sections from 3-month-old mice showed strong Reelin positivity that was mainly located extracellularly (Fig. 2j–o). Only weak intracellular Reelin labeling was detected in Calbindin D-28k^+^ neurons (asterisks in Fig. 2j–o). These results demonstrate that robust Reelin expression is present in the striatum at various developmental stages ranging from E16 to 3 months. Although, at P0, distinct expression of Reelin is detectable in the midbrain, our results suggest that the main sources of Reelin are the striatal inhibitory neurons.

### VLDLr localization in mouse midbrain and striatum

**Fig. 3:**
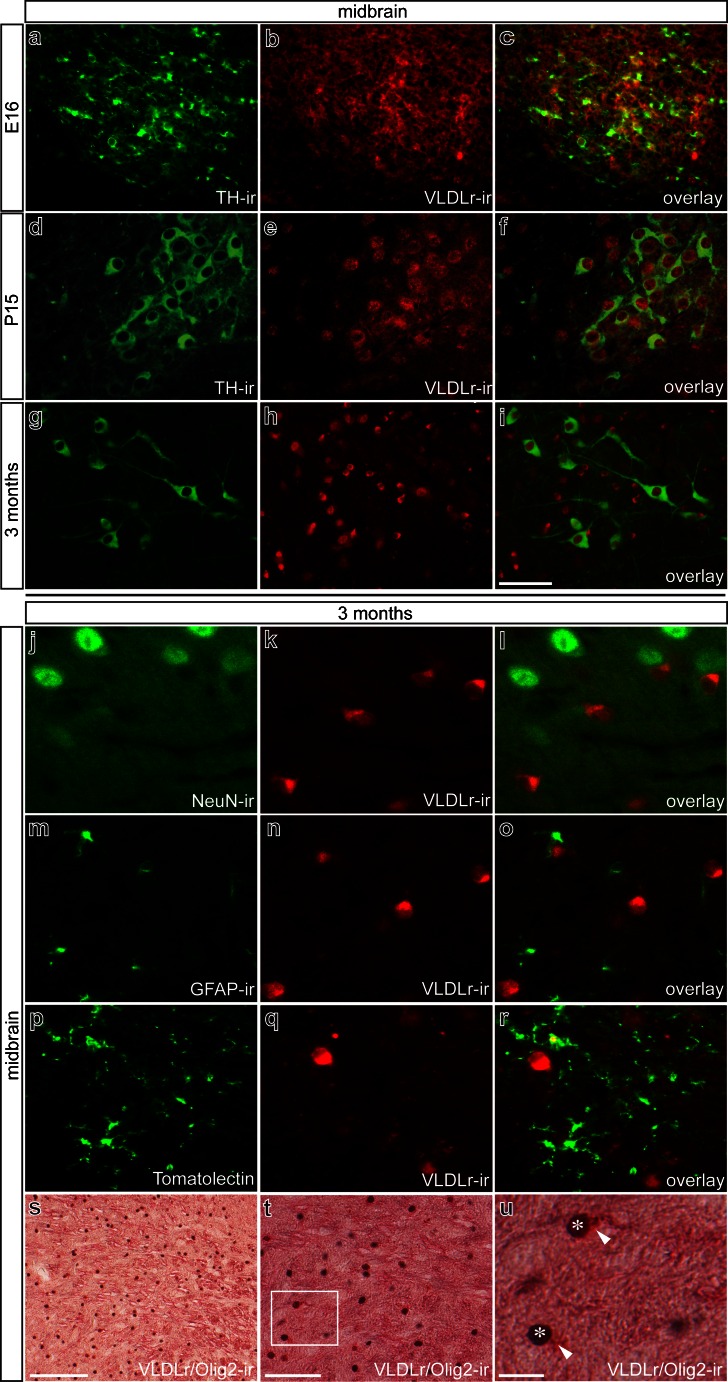
Very low density lipoprotein receptor (*VLDLr*) distribution pattern in mouse midbrain in embryonic, postnatal, and adult stages. **a–c** Double-immunofluorescence for the dopaminergic marker TH (**a**) and VLDRr (**b**) in fixed midbrain sections of mouse at E16. Overlay (**c**) illustrates expression of VLDLr in dopaminergic neurons. **d–f** Double-immunofluorescence for TH (**d**) and VLDRr (**e**) in substantia nigra at P15 reveals co-localization of the proteins (**f**). **g–i** In 3-month-old mice, although VLDLr immunoreactivity is present in ventral midbrain (**h**), dopaminergic neurons are devoid of VLDLr expression (**i**). **j–u** Characterization of midbrain cells expressing VLDLr in 3-month-old mice. Double-immunofluorescence for VLDLr and neuronal nuclear antigen (*NeuN*; **j–l**), the astrocyte marker glial fibrillary acidic protein (*GFAP*; **m–o**), or Tomatolectin (**p–r**) reveals that VLDLr is not colocalized with these proteins. **s**, **t** Overviews of double immunohistochemistry for VLDRr and oligodendrocyte marker Olig2. **t** Co-localization of VLDLr and Olig2 proteins. **u** Higher magnification of *white boxed area* in **t** illustrating nuclear Olig2 labeling (*asterisks*) and intracellular VLDLr labeling (*arrowheads*). *Bars* 100 μm (**s**), 50 μm (**a–i**, **t**), 20 μm (**j–r**, **u**)

To examine further the localization pattern of VLDLr receptors in midbrain mDA neurons and in the striatum, we conducted double-immunofluorescence staining by using a rabbit polyclonal antibody specific to the amino-terminal domains of VLDLr at various embryonic and postnatal stages. As shown in Fig. 3a–f, VLDLr expression was found in TH^+^ mDA neurons and to a lesser extent in adjacent neuron populations. At 3 months, TH^+^ neurons showed no positivity for VLDLr, whereas several small-sized cells in close proximity to TH^+^ neurons displayed strong VLDLr signals (Fig. 3g–i). In order to identify these cells, we used several cellular markers. Figure 3j–l demonstrates that VLDLr was not expressed by midbrain neurons (positive for neuronal nuclear antigen [NeuN^+^]) at 3 months. Moreover, no glial fibrillary acidic protein (GFAP)^+^ astrocytes (Fig. 3m–o), Tomatolectin^+^ microglia, or endothelial cells (Fig. 3p–r) were positive for VLDLr. Using VLDLr in combination with Olig2 as a marker for oligodendrocytes, we were able to show that oligodendroglia cells express VLDLr in midbrains of 3-month-old mice (Fig. 3s–u).

**Fig. 4:**
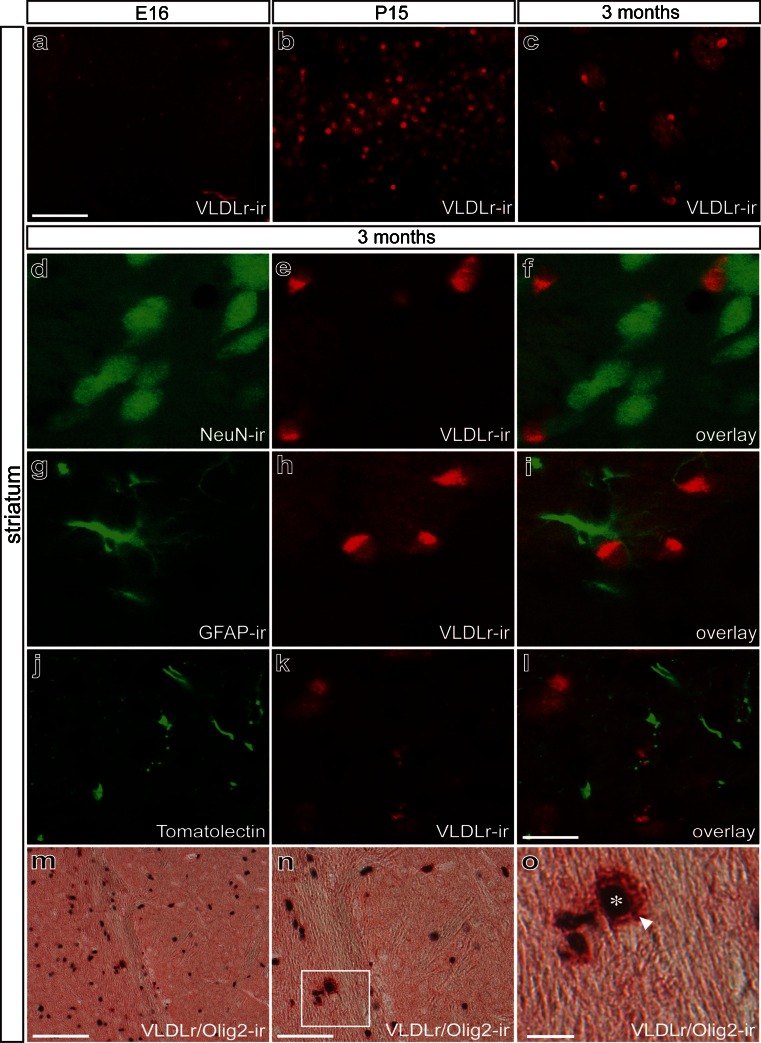
Localization of VLDLr in mouse striatum in embryonic, postnatal, and adult stages. **a–c** at E16 (**a**), no VLDLr immunoreactivity is detected, whereas at P15 (**b**), VLDLr expression is prominent and declines again at 3 months (**c**). **d-o** Identification of striatal cells expressing VLDLr at 3 months. Double-immunofluorescence with NeuN (**d–f**), GFAP (**g–i**), and Tomatolectin (**j–l**) illustrates no colocalization of the proteins. **m–o** Double-immunohistochemistry for the oligodendrocyte marker Olig 2 and VLDLr reveals co-expression of the proteins. **o** Higher magnification of the *white boxed area* in **n**. Strong intracellular VLDLr labeling (*arrowhead*) is shown in Olig2-expressing cells (*asterisk*). *Bars* 100 μm (**m**), 50 μm (**a–c**, **n**), 20 μm (**d–l**, **o**)

Analysis of VLDLr expression in the striatum revealed virtually no expression at E16, strong expression at P15, and intense expression that was restricted to a small subset of cells at 3 months (Fig. 4a–c). Figure 4d–f clearly demonstrates that several NeuN^+^ striatal neurons did not express VLDLr at 3 months. Further, GFAP^+^ astrocytes (Fig. 4g–i) and Tomatolectin^+^ microglia (Fig. 4j–l) were not VLDLr^+^ at 3 months. As described for the midbrain, Olig2^+^ oligodendroglia showed strong immunoreactivity for VLDLr, demonstrating that the oligodendroglia are the cell population expressing VLDLr in the striatum at 3 months of age (Fig. 4m–o).

**Fig. 5:**
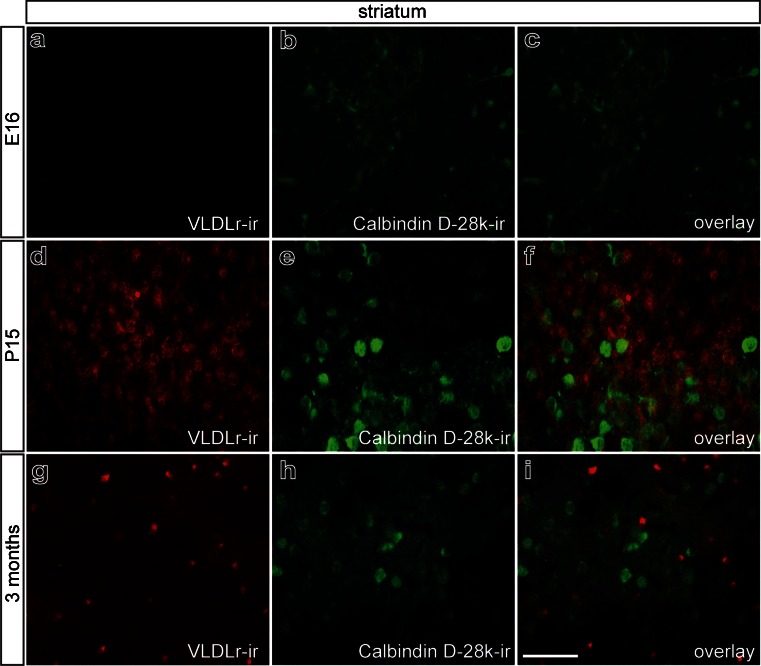
Identification of striatal cells expressing VLDLr. Double-immunofluorescence for VLDLr and Calbindin D-28k. At E16 (**a–c**), VLDL-r expression is absent in mouse striatum. At P15 (**d–f**), striatal Calbindin D-28k^+^ cells also express VLDLr. At 3 months (**g–i**), no colocalization is observed between Calbindin D-28k and VLDL. *Bar* 50 μm

In order to identify the neuronal subpopulation that expresses VLDLr in the striatum, double-labeling for VLDLr and Calbindin D-28k was performed at E16, P15, and 3 months. As shown in Fig. 5, striatal Calbindin D-28k^+^ neurons only expressed VLDLr at P15 (Fig. 5d–f). No VLDLr expression was detectable at E16 (Fig. 5a–c), and at 3 months (Fig. 5g–i), VLDLr expression occurred neither in Calbindin D-28k^+^ neurons nor in any other neuron population.

Taken together, these results demonstrate that the expression of VLDLr was present at early postnatal stages in mDA midbrain neurons and in Calbindin D-28k^+^ striatal inhibitory neurons. At 3 months, VLDLr expression is restricted to oligodendrocytes.

### ApoER2 expression in murine midbrain and striatum

**Fig. 6:**
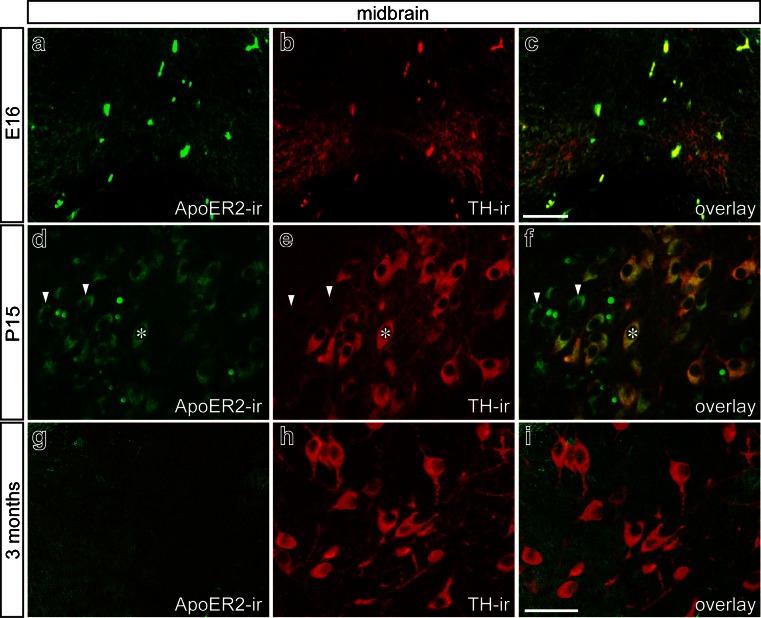
Apolipoprotein E receptor type 2 (*ApoER2*) distribution pattern in mouse midbrain in embryonic, postnatal, and adult stages. Double-immunofluorescence for the dopaminergic marker TH and ApoER2 on fixed midbrain sections of mouse at E16 (**a–c**), at P15 (**d–f**), and in 3-month-old mice (**g–i**). At E16, mDA neurons express ApoER2 (*overlay* in **c**). At P15 (**d–f**), TH-immunoreactive neurons of the substantia nigra also reveal ApoER2 immunoreactivity (*asterisks* a cell that exhibits both TH and ApoER2 immunolabeling, *arrowheads* cells positive for ApoER2 but negative for TH). In 3-month-old mice (**g–i**), no ApoER2 immunoreactivity was detectable in midbrain. *Bars* 50 μm

The expression of the Reelin receptor ApoER2 was analyzed at E16, P15, and 3 months in the midbrain (Fig. 6). At E16, virtually all TH^+^ midbrain neurons were positive for ApoER2 (Fig. 6a–c). Analysis of TH^+^ neurons from the substantia nigra revealed that all mDA neurons expressed ApoER2 at P15 (asterisks in Fig. 6d–f). However, several cells not labeled by TH-immunostaining (Fig. 6e, arrowheads) displayed strong ApoER2 immunoreactivity (Fig. 6d, f). Interestingly, no ApoER2 expression was detectable in the midbrain at 3 months of age (Fig. 6g–i).

**Fig. 7:**
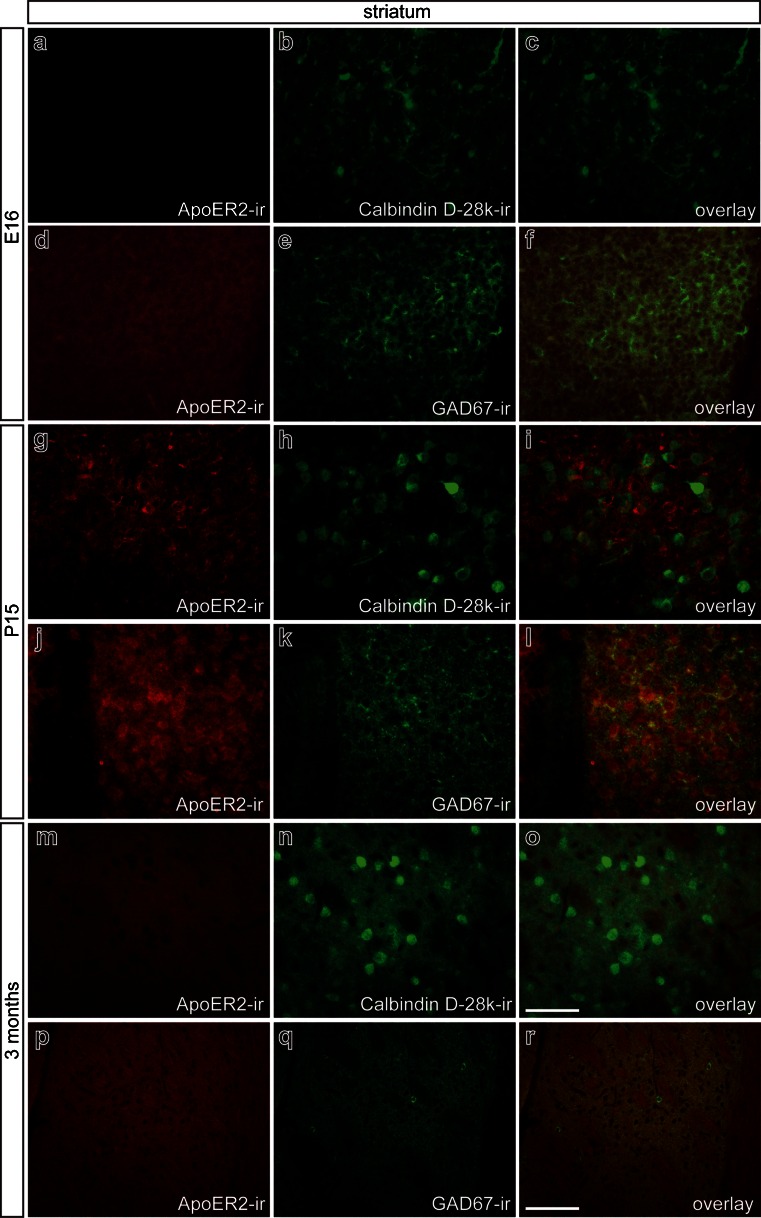
Localization of ApoER2 in mouse striatum in embryonic, postnatal, and adult stages. Double-immunofluorescence for ApoER2 with Calbindin D-28k and glutamic acid decarboxylase 67 (*GAD67*) at E16 (**a–f**), at P15 (**g–l**), and 3 months (**m–r**). Overlays for ApoER2 and Calbindin D-28k reveal that Calbindin D-28k-immunopositive cells are devoid of ApoER2 at E16 and at 3 months. In contrast, at P15, both Calbindin D-28k^+^ neurons and other cells express ApoER2 (**i**). Overlays for ApoER2 and GAD67 reveal that GAD67-immunopositive cells are devoid of ApoER2 at E16 (**f**) and at 3 months (**r**). In contrast, at P15, a subset of ApoER2-immunoreactive cells also express GAD67 (**l**). *Bars* 50 μm

Analysis of ApoER2 expression in the striatum at E16, P15, and 3 months revealed that ApoER2 immunoreactivity was present at P15 (Fig. 7g–l). At E16 (Fig. 7a–f) and 3 months (Fig. 7m–r), no ApoER2 signal was observed. Using Calbindin D-28k (Fig. 7h, i) and GAD67 (Fig. 7k, l) as markers for inhibitory striatal interneurons, we were able to show that ApoER2 was expressed by these striatal neuron populations. Notably, GAD67^+^ striatal neurons showed a stronger ApoER2 signal. However, some neurons expressing ApoER2 were not positive for the abovementioned markers and, thus, represented another striatal neuron population. Using acetylcholinesterase as a marker, we were able to identify these cells as cholinergic interneurons (data not shown). Together, these data indicate that the expression of ApoER2 in midbrain and striatum is restricted to early postnatal stages and is no longer observed in the mature mesostriatal and mesocorticolimbic system.

### Dab1 expression in mouse midbrain and striatum

**Fig. 8:**
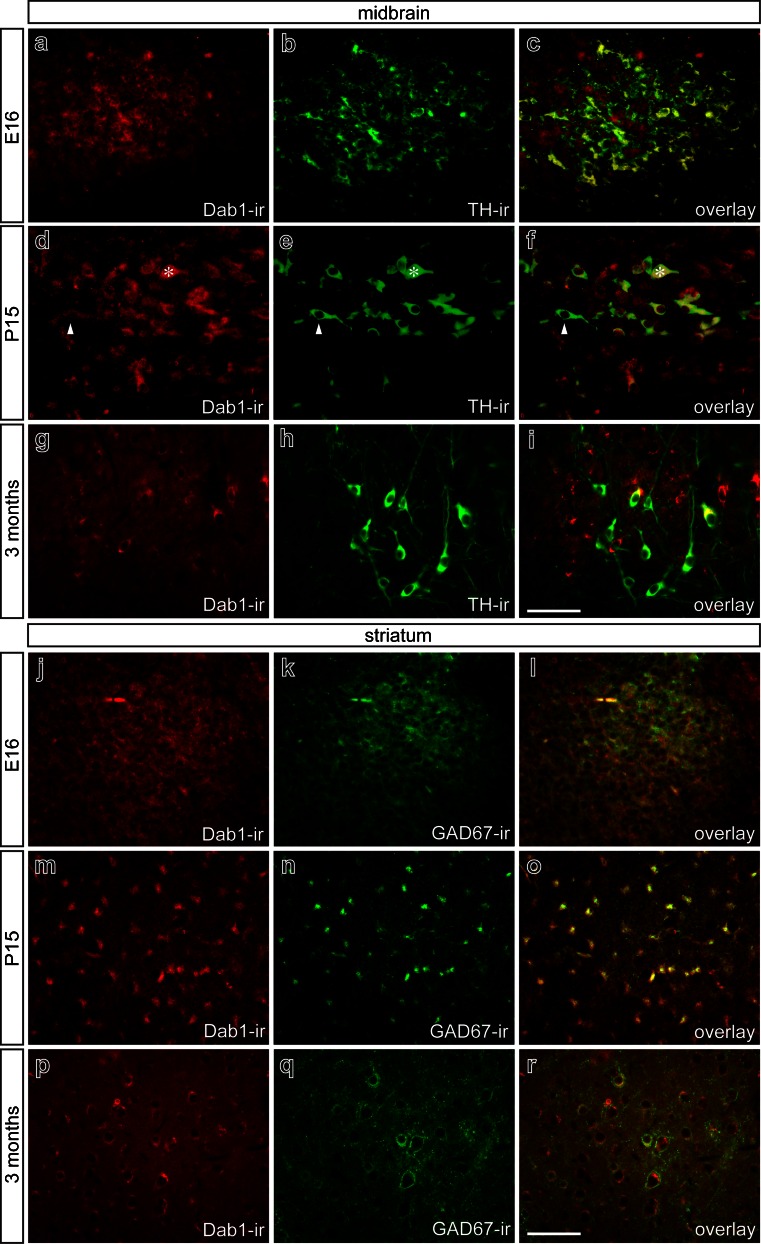
Disabled adaptor protein (*Dab1*) distribution pattern in mouse midbrain and striatum in embryonic, postnatal, and adult stages. **a–i** Double-immunofluorescence for Dab1 and TH in mouse midbrain at E16 (**a–c**), P15 (**d–f**), and 3 months (**g–i**) Overlays **c**, **f**, **i** illustrate Dab1 expression in dopaminergic neurons at E16, P15, and 3 months (*asterisks* TH^+^ neuron with high Dab1 expression, *arrowheads* TH^+^ neuron with low Dab1 expression). In mouse striatum **j–r**), Dab1 immunoreactivity is observed at E16 (**j–l**), P15 (**m–o**), and 3 months (**p–r**) and is co-localized with GAD67. *Bars* 50 μm

Dab1, the essential intracellular downstream mediator for Reelin signaling, was expressed by TH^+^ midbrain neurons at E16 (Fig. 8a–c), P15 (Fig. 8d–f), and to a lesser extent, 3 months (Fig. 8g–i). However, TH^+^ neurons were not the only cell population expressing Dab1, since several neurons in close proximity to TH^+^ neurons also showed clear immunoreactivity for Dab1, which was most pronounced at 3 months (Fig. 8g–i). Moreover, TH^+^ neurons expressed Dab1 at different levels. Especially at P15, we identified TH^+^ neurons with high Dab1 expression (asterisks in Fig. 8d–f) and neurons with low Dab1 expression (arrowheads in Fig. 8d–f). Analysis of Dab1 expression in the striatum at E16 (Fig. 8j–l), P15 (Fig. 8m–o), and 3 months (Fig. 8p–r) revealed that Dab1 was expressed at all time points analyzed. Using GAD67 as a marker, we were able to demonstrate that GABAergic interneurons expressed Dab1 (Fig. 8l, o, r). These data demonstrate that Dab1 shows a more distinct expression pattern in midbrain and striatum than the Reelin receptors VLDLr and ApoER2.

### APP expression in the mouse midbrain and striatum

**Fig. 9:**
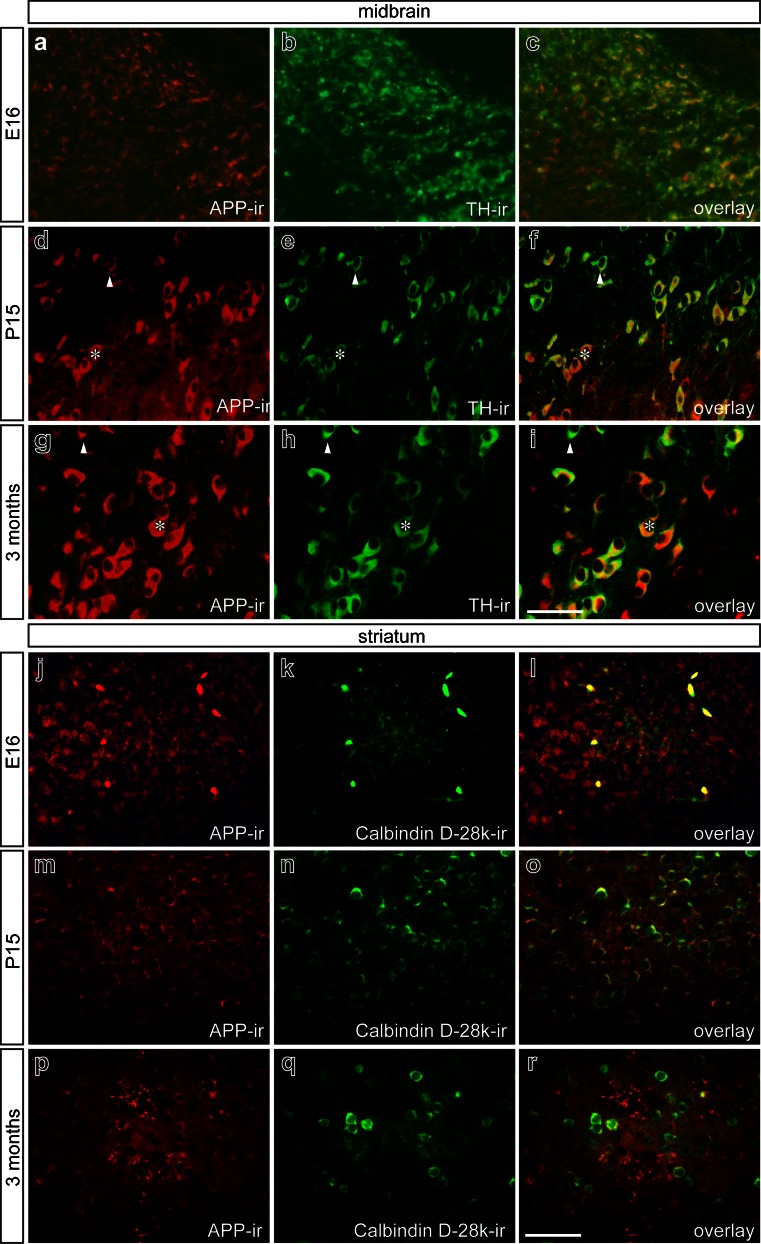
Amyloid beta precursor protein (*APP*) distribution pattern in mouse midbrain and striatum in embryonic, postnatal, and adult stages. Double-immunofluorescence for APP and TH in mouse midbrain at E16 (**a–c**), P15 (**d–f**), and 3 months (**g–i**). Overlays **c**, **f**, **i** illustrate APP expression in dopaminergic neurons at E16, P15, and 3 months (*asterisks* TH^+^ neuron with high APP expression, *arrowheads* TH^+^ neuron with low APP expression). In mouse striatum (**j–r**), APP abundance is prominent at E16 (**l**) and declines at P15 (**o**) and at 3 months (**r**). Double-immunofluorescence reveals colocalization of APP with Calbindin D-28k. *Bars* 50 μm

APP, which is related to the LDL family of proteins (Siemes et al. 2006; Young-Pearse et al. 2007; Hoareau et al. 2008), has been shown to be an alternative receptor for Reelin (Hoe et al. 2009). Therefore, we further analyzed the expression pattern of APP in the murine midbrain and striatum at E16, P15, and 3 months. As depicted by Fig. 9, APP immunoreactivity was observed at E16 (Fig. 9a–c), P15 (Fig. 9d–f), and 3 months (Fig. 9g–i) and was predominantly found in TH^+^ midbrain neurons. Overlay images (Fig. 9c, f, i) suggest that only a subset of TH^+^ neurons expressed APP. However, this impression was attributable to the different expression levels of APP as indicated by the asterisks (high APP expression) and arrowheads (low APP expression) in Fig. 9d–i. Analysis of APP expression in the striatum revealed APP immunoreactivity at E16 (Fig. 9j–l), P15 (Fig. 9m–o), and 3 months (Fig. 9p–r). Using Calbindin D-28k as a marker, we were able to demonstrate that striatal inhibitory neurons robustly expressed APP at all time points analyzed (Fig. 9l, o, r).

In summary, TH^+^ midbrain neurons and striatal inhibitory neurons express APP from embryonic stages into adulthood, further suggesting a role for APP in the mesostriatal and mesocorticolimbic system extending beyond developmental processes.

## Discussion

In the present study, we have elucidated the temporal and spatial expression patterns of Reelin and its signaling receptors VLDLr and ApoER2. Moreover, we have further analyzed the expression of the intracellular downstream mediator Dab1 and the alternative Reelin receptor APP in the murine midbrain and striatum. The development of mDA neurons is a complex process that involves the early specification and phenotype induction of this neuron population plus the essential migration event of young mDA neurons to give rise to the anatomical specified midbrain regions VTA and SN. Newborn TH^+^ neurons of the midbrain, which later become SN neurons, have to migrate from the ventral floor in a radial manner and must further migrate tangentially to be positioned laterally in the SN pars compacta (Smidt and Burbach 2007). The initial evidence for a functional role of Reelin during the migration of SN neurons was presented by Nishikawa and colleagues (2003). The authors demonstrated that Reelin mutant mice showed an accumulation of SN neurons lateral to the VTA because of a migration failure of these neurons. Interestingly, the fiber projections of the misplaced mDA neurons were not impaired, and the proper innervation of the striatum was observed. A similar migratory phenotype of mDA neurons, which are destined to become SN neurons, has also been described in ApoER2^−/−^, VLDLr^−/−^, and ApoER2/VLDLr double-mutant mice and in mice lacking the intracellular Reelin downstream mediator Dab1 (Sharaf et al. 2013; Kang et al. 2010). These studies clearly demonstrate the important roles of Reelin and its signaling components in the migration and proper positioning of the mDA neurons of the SN. However, a detailed analysis of the expression patterns of Reelin and its receptors in the mouse midbrain and striatum has, until now, been missing. Here, we describe the expression patterns of Reelin, VLDLr, ApoER2, Dab1, and APP in the mouse midbrain and striatum at E16, P15, and 3 months of age. We have clearly demonstrated that the Reelin receptors ApoER2 and VLDLr are expressed by TH^+^ midbrain neurons at E16 and P15 but not at 3 months. The expression pattern of the aforementioned Reelin signaling components in the striatum is different. At P15, Calbindin D-28k^+^/GAD67^+^ inhibitory spiny neurons express ApoER2 and VLDLr. Interestingly, no expression of ApoER2 and VLDLr is observed in striatal neurons at E16 or 3 months. Notably, the expression of VLDLr in the midbrain and striatum at 3 months is restricted to the oligodendrocytes and is no longer detectable in neurons. Myelinating oligodendrocytes have been reported to express LDLr and VLDLr selectively in the postnatal spinal cord at P7 and P15 (Zhao et al. 2007), and the VLDLr^+^ oligodendrocytes observed in both midbrain and striatum might also be involved in myelination processes.

The temporal expression patterns of ApoER2 and VLDLr underline the importance of Reelin signaling during embryonic development and the early postnatal maturation of the mesostriatal and mesocorticolimbic system und further suggest that Reelin signaling is not involved in promoting the maintenance and survival of TH^+^ neurons in the mature system. Studies of mice mutant for Reelin receptors further support this hypothesis, since none of the mutants analyzed show any substantial loss of midbrain TH^+^ neurons. The reduced numbers of TH^+^ neurons in the SN of these mutants arises because of a lack of migration and the subsequent clustering of SN neurons at the lateral border of the VTA. A reduction in total numbers of midbrain TH^+^ neurons has not been detected (Kang et al. 2010; Sharaf et al. 2013). These data provide evidence that Reelin signaling is dispensable for neuron survival but indispensable for the migration and positioning of TH^+^ neurons of the SN, as is further supported by a recent study by Bodea and colleagues (2014). The authors have demonstrated that the distinct migratory pathways taken by VTA and SN neurons are determined by specific signaling pathways. Whereas exclusively radially migrating VTA neurons require CXCR4/CXCL12 signaling, tangentially migrating SN neurons are dependent on ApoER2/VLDLr/Reelin signaling. The finding that Dab1^−/−^ mice show the same phenotype as the mice mutant for Reelin receptors indicates the importance of Dab1 as the essential intracellular downstream mediator of Reelin signals (Kang et al. 2010; Sharaf et al. 2013). Notably, we have observed a level of Dab1 expression that exceeds that of ApoER2 and VLDLr. At 3 months, Dab1 is detectable in TH^+^ neurons and in GAD67^+^ striatal neurons. This expression pattern is similar to the pattern observed for the alternative Reelin receptor APP, which shows strong expression in TH^+^ neurons and striatal neurons at 3 months. Interestingly, APP has been demonstrated to be able to interact with and activate Dab1 (Homayouni et al. 1999). The functional significance of these interactions is unclear, but we have to take into account that Dab1 might also be involved in pathways independent of Reelin. A contribution to neurodegenerative processes in the mesostriatal and mesocorticolimbic system should be considered, especially in the context of the APP-Dab1 axis. Mice lacking APP have been shown to develop attenuated degeneration of mDA neurons following the axotomy of the medial forebrain bundle (DeGiorgio et al. 2002a) accompanied by decreased microglia activation and enhanced neuron survival (DeGiorgio et al. 2002b).

In the present study, we have further addressed the expression of Reelin in the mouse midbrain and striatum at E16, P15, and 3 months. Distinct Reelin expression in midbrain neurons could only be detected at P0, and extremely weak Reelin immunoreactivity was observed at 3 months. However, in the striatum, we could observe continuous Reelin expression from E16 to 3 months. Interestingly, Reelin was predominantly located intracellularly at E16 and could be detected intracellularly and extracellularly at P15. Moreover, at 3 months, Reelin was barely detectable in the cytoplasm of striatal neurons and seemed to be predominantly located extracellularly. These results suggest that the striatum and not the midbrain is the primary source of Reelin for midbrain neurons, and that Reelin is transported retrogradely via fibers of TH^+^ neurons. However, Nishikawa and colleagues (2003) have demonstrated that anterograde Reelin transport via the axonal projections of embryonic striatal neurons takes place. To our knowledge, a retrograde transport of Reelin from the striatum to the midbrain by mDA neurons has not as yet been shown. Thus, we need to establish whether retrograde transport of Reelin via dopaminergic axons takes place and is involved in the Reelin-mediated positioning of TH^+^ neurons.

Taken together, the observed expression patterns of Reelin and its signaling components in the embryonic and postnatal midbrain and striatum presented in the present study reflect the importance of the Reelin signaling pathway during embryonic and early postnatal development. The loss of ApoER2 and VLDLr expression in the mature midbrain and striatum demonstrates that Reelin functions are restricted to migratory events and early postnatal maturation and are dispensable for the maintenance of TH^+^ neurons. The expression pattern data presented in this study provide the basis for an understanding of Reelin signaling-mediated mechanisms during the migration of the TH^+^ neurons of the mesostriatal and mesocorticolimbic system.
